# Supplementary material for: Acceleration of western Arctic sea ice loss linked to the Pacific North American pattern
Source: Nat Commun. 2021 Mar 9;12:1519. doi: 10.1038/s41467-021-21830-z (PMC7943814; doi:10.1038/s41467-021-21830-z)
Supplement: Supplementary file 1 — Supplementary information [file 41467_2021_21830_MOESM1_ESM.pdf]

Acceleration of western Arctic sea ice loss linked to the Pacific North American pattern

Zhongfang Liu[1*], Camille Risi[2], Francis Codron[3], Xiaogang He[4], Christopher J. Poulsen[5], Zhongwang Wei[6,7], Dong Chen[8], Sha Li[9] and Gabriel J. Bowen[10]

[1] State Key Laboratory of Marine Geology, Tongji University, Shanghai 200092, China

[2] Laboratoire de Météorologie Dynamique, IPSL, CNRS, Sorbonne Université, Paris 75252, France

[3] LOCEAN, Sorbonne Université, Paris 75252, France

[4] Department of Civil and Environmental Engineering, National University of Singapore, 117576, Singapore

[5] Department of Earth and Environmental Sciences, University of Michigan, Ann Arbor, MI 48109, USA

[6] Guangdong Province Key Laboratory for Climate Change and Natural Disaster Studies, School of Atmospheric Sciences, Sun Yat-sen University, Guangzhou 510275, China

[7] Southern Marine Science and Engineering Guangdong Laboratory (Zhuhai), Zhuhai 519000, China

[8] Nansen-Zhu International Research Centre, Institute of Atmospheric Physics, Chinese Academy of Sciences, Beijing 100029, China

[9] Department of Earth System Science, Tsinghua University, Beijing 100084, China

[10] Department of Geology and Geophysics, University of Utah, Salt Lake City, UT 84112, USA

* Corresponding author: Zhongfang Liu, email: liuzf406@gmail.com

This document includes the Supplementary Figures that are referred to in the main text.

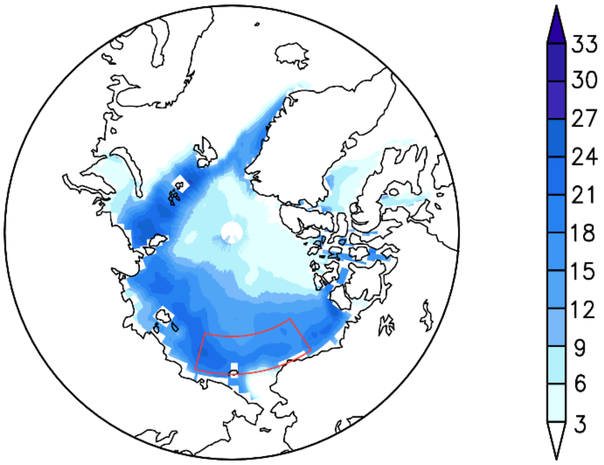

Supplementary Figure 1 | Interannual to decadal variability in summer Arctic SIC. The variability is defined by the standard deviation of summer mean SIC over the period 1979–2016. The box marks the western Arctic Ocean defined in Fig. 1.

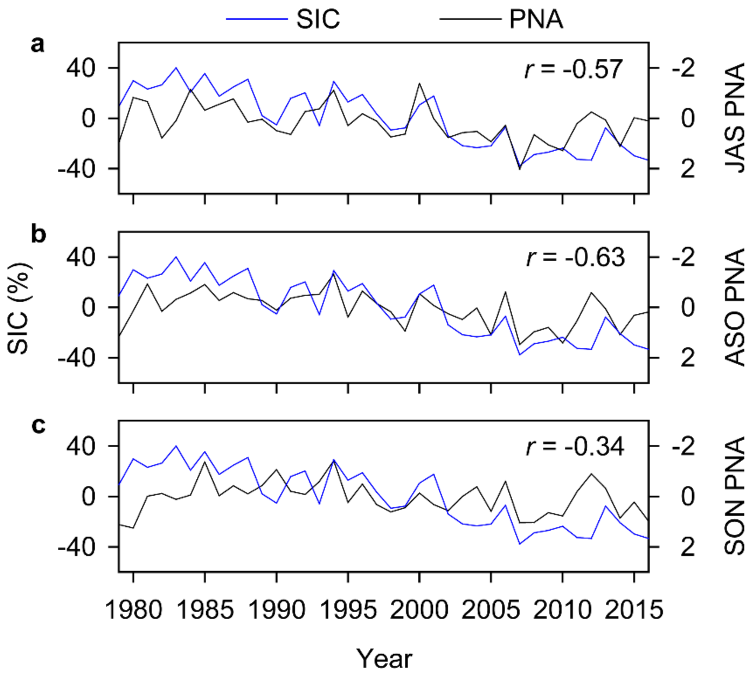

Supplementary Figure 2 | Lead-lag relationship between the PNA and western Arctic SIC indices. a–c, The SIC index lags the PNA index by 1-month (**a**), 0-month (**b**) and -1-month (**c**). The values on the plots show the correlation coefficients and all are significant at the 5% level.

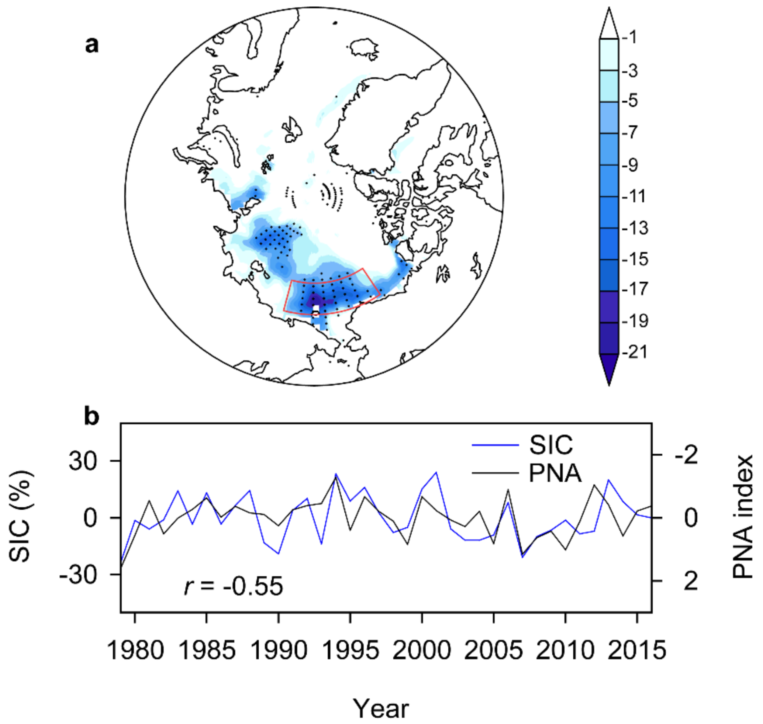

Supplementary Figure 3 | Arctic SIC responses to interannual PNA variability. a, Detrended SIC regressed onto the PNA index. The stippling indicates statistical significance at the 5% level and the box marks the western Arctic Ocean defined in Fig. 1. **b**, Detrended time series of the PNA and western Arctic SIC indices.

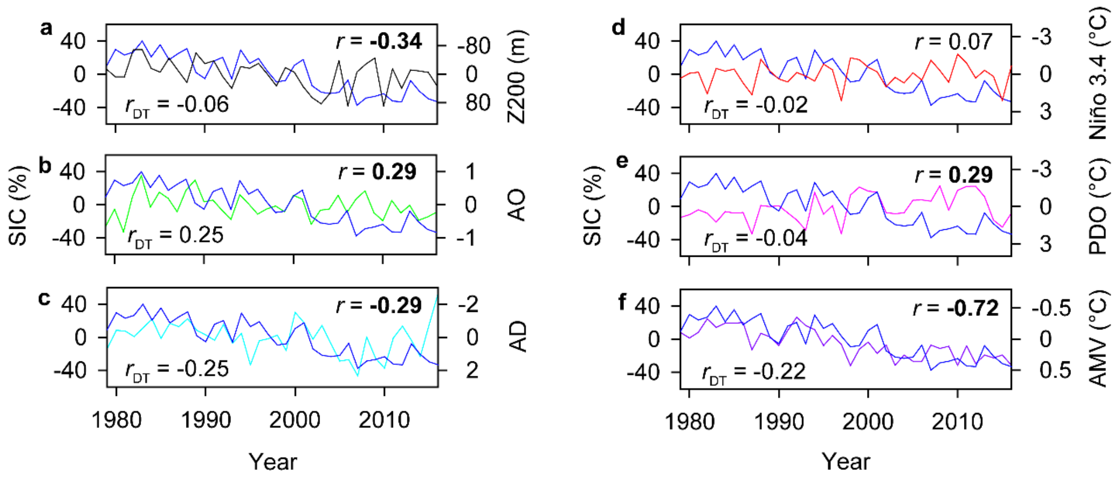

Supplementary Figure 4 | Relationship between the western Arctic SIC and climate indices. a, Time series of western Arctic SIC and anticyclonic circulation over Greenland (GL-Z200) defined by Ref[1]. **b–f**, Same as **a** but for the North Atlantic Oscillation (NAO, **b**), the Arctic Dipole (AD, **c**), the Niño 3.4 (**d**), the Pacific Decadal Oscillation (PDO, **e**) and the Atlantic Multidecadal Variability (AMV, **f**). r and r_{DT} denote the non-detrended and detrended correlations, respectively, and bold type indicates statistical significance at the $p < 0.1$ level.

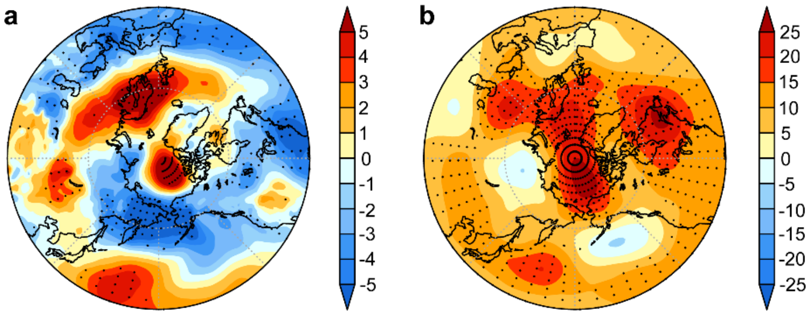

Supplementary Figure 5 | Atmospheric circulation patterns linked to western Arctic SIC changes. a, 1000 hPa geopotential height (m) regressed onto the standardized western Arctic SIC index. **b**, Same as **a** but for 250 hPa geopotential height (m). The stippling indicates statistical significance at the 5% level.

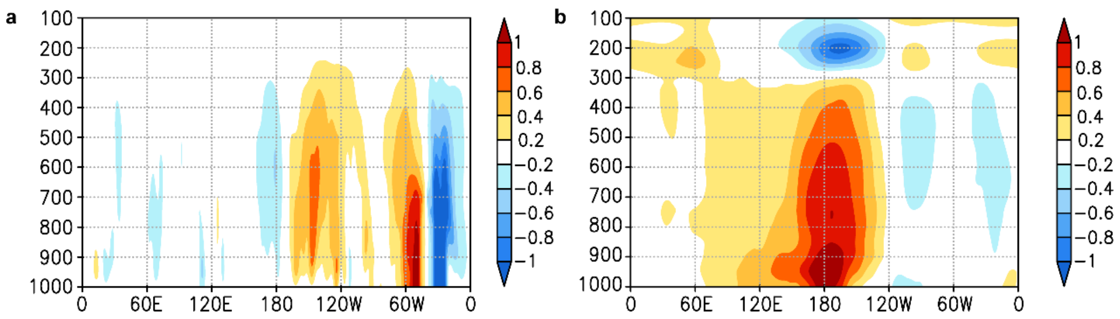

Supplementary Figure 6 | Vertical velocity and temperature responses to interannual PNA variability. a, Height–longitude cross sections of vertical velocity (10^{-2} Pa s^{-1}) averaged over 70–80°N regressed onto the PNA index. **b**, Same as **a** but for temperature (°C). All climate variables were linearly detrended before the calculation.

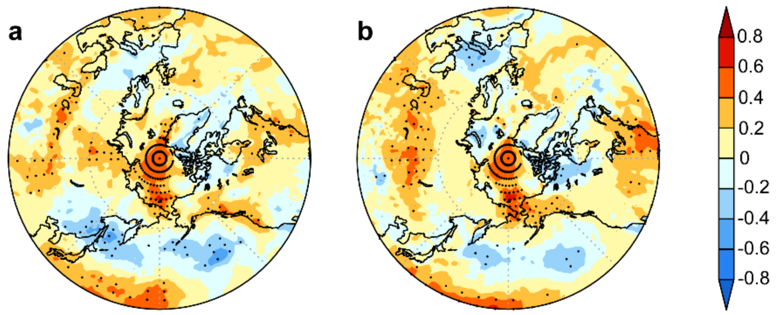

Supplementary Figure 7 | Radiation responses to interannual PNA variability. a, Spatial correlations of the PNA index with downwelling longwave radiation (DLR). **b,** Same as **a** but for upwelling longwave radiation (ULR). All climate variables were linearly detrended before the calculation. The stippling indicates statistical significance at the 5% level in all plots.

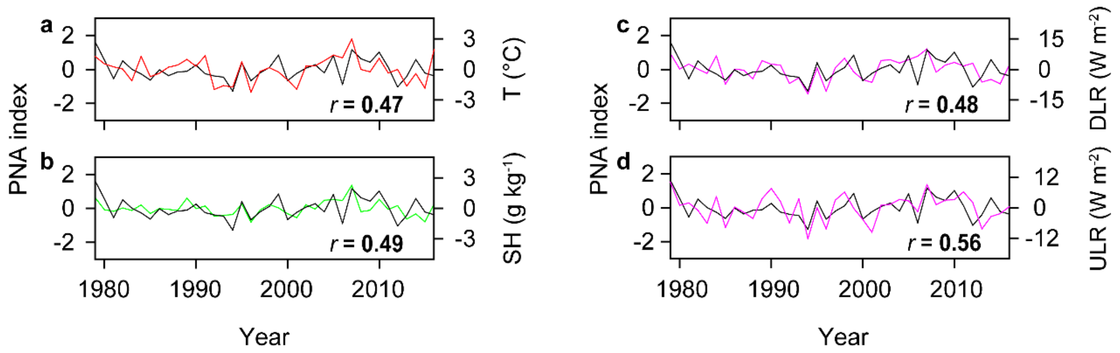

Supplementary Figure 8 | Relationship between the PNA index and western Arctic thermodynamic variables. a, Time series of the detrended PNA index and lower-tropospheric (1000–850 hPa) temperature (T). **b**–**d**, Same as **a** but for lower-tropospheric specific humidity (SH, **b**), downwelling longwave radiation (DLR, **c**) and upwelling longwave radiation (ULR, **d**). The values on the plots show the correlation coefficients, and bold type indicates statistical significance at the 5% level.

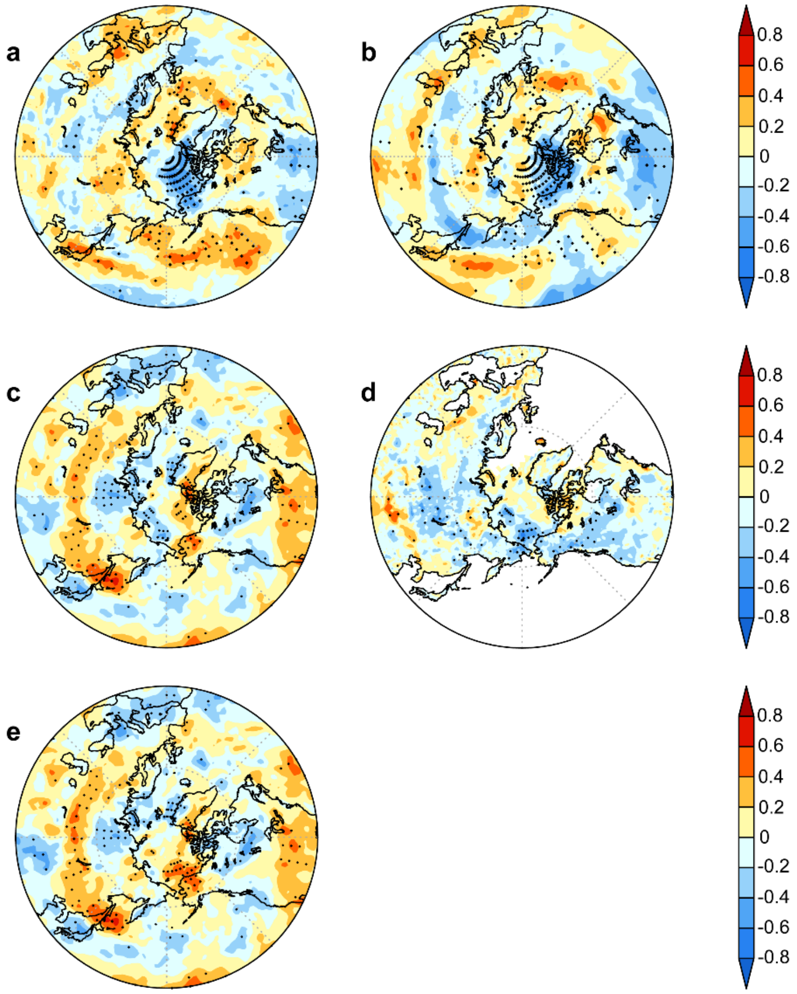

Supplementary Figure 9 | Radiation responses to interannual PNA variability. a, Spatial correlations of the PNA index with middle cloud cover (MCC). **b–e,** Same as **a** but for high cloud cover (HCC, **b**), downwelling shortwave radiation (DSR, **c**), surface albedo (AL, **d**) and net shortwave radiation (NSR, **e**). All climate variables were linearly detrended before the calculation. The stippling indicates statistical significance at the 5% level in all plots.

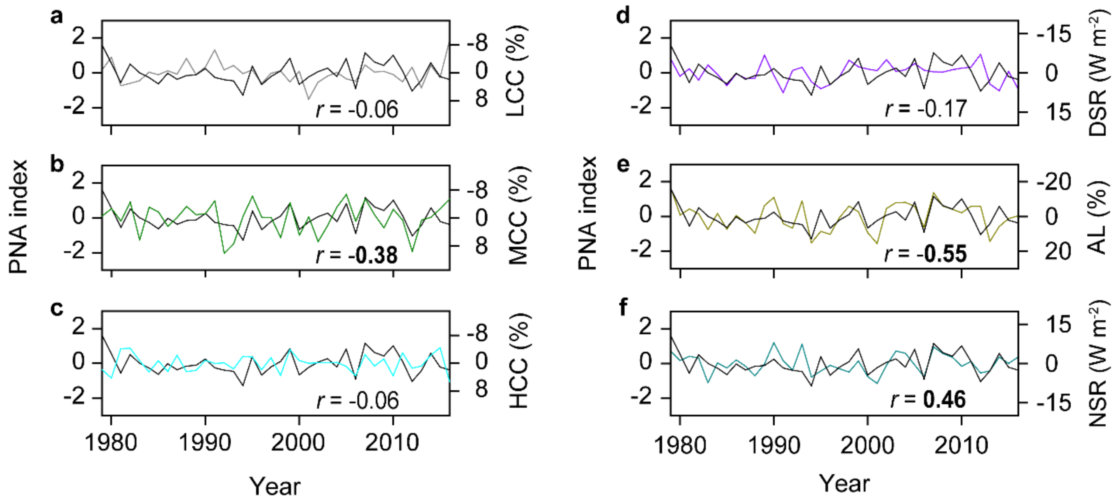

Supplementary Figure 10 | Relationship between the PNA index and western Arctic radiation variables. a, Time series of the detrended PNA index and western Arctic low cloud cover (LCC). **b–f**, Same as **a** but for middle cloud cover (MCC, **b**), high cloud cover (HCC, **c**), downwelling shortwave radiation (DSR, **d**), surface albedo (AL, **e**) and net shortwave radiation (NSR, **f**). The values on the plots show the correlation coefficients, and bold type indicates statistical significance at the 5% level.

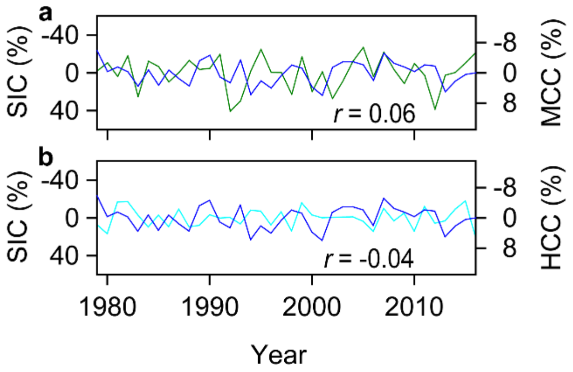

Supplementary Figure 11 | Relationship between western Arctic SIC and cloud cover. a, b, Time series of the detrended western Arctic SIC, middle cloud cover (MCC, **a**) and high cloud cover (HCC, **b**). The values on the plots show the correlation coefficients.

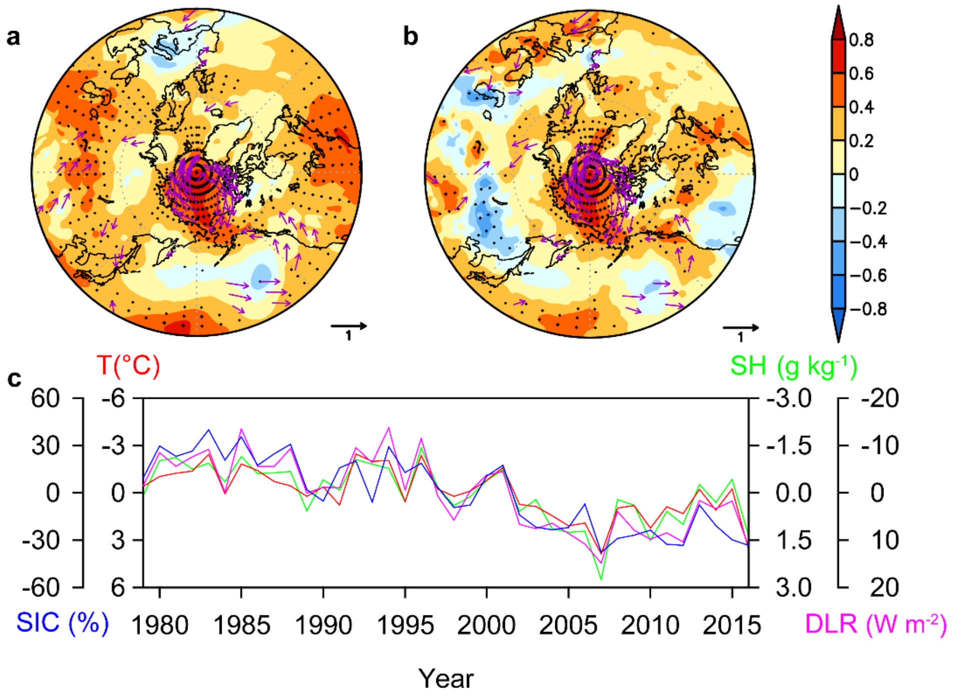

Supplementary Figure 12 | PNA-induced thermodynamic changes and its influence on western Arctic SIC. a, Spatial correlations between the non-detrended PNA index and western Arctic lower-tropospheric temperature (shading) and vertically integrated heat flux (arrows). **b**, Same as **a** but for lower-tropospheric specific humidity (shading) and vertically integrated moisture flux (arrows). **c**, Time series of the non-detrended western Arctic SIC index (blue) and the lower-tropospheric temperature (T, red) humidity (SH, green) and downwelling longwave radiation (DLR, pink). Shading with stippling in **a** and **b** indicates statistical significance at the 5% level.

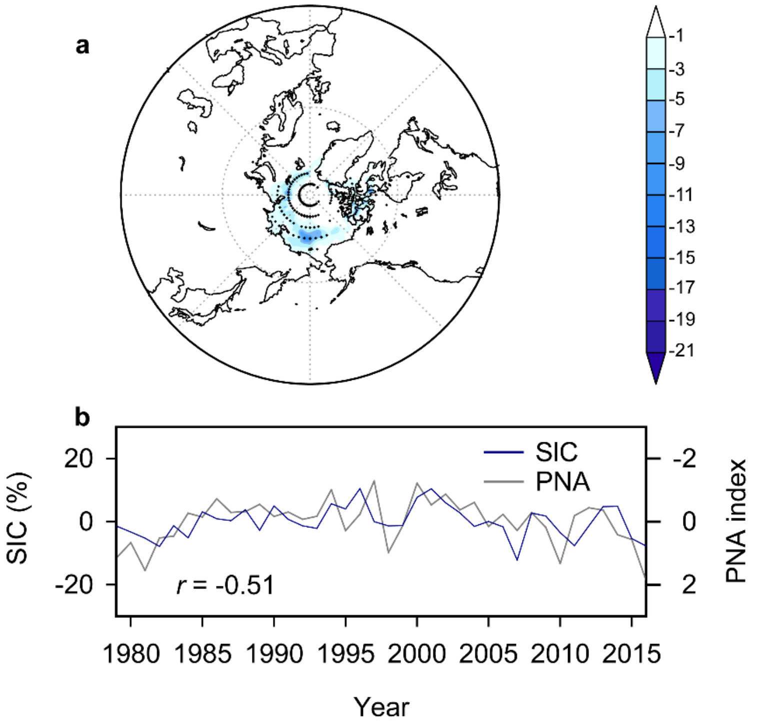

Supplementary Figure 13 | Simulated Arctic SIC responses to interannual PNA variability. a, Detrended SIC regressed onto the PNA index. **b**, Detrended time series of the PNA and SIC indices. The stippling indicates statistical significance at the 5% level in **a**.

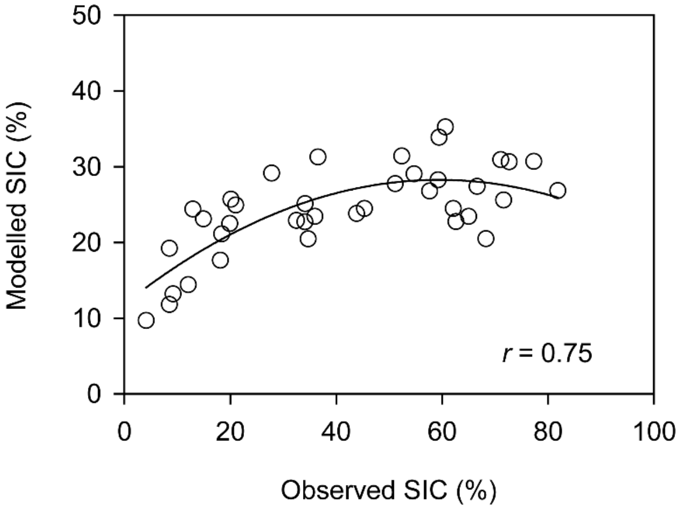

Supplementary Figure 14 | Comparison between the observed and simulated western Arctic SIC. The black line is a second-order polynomial fit to the data with a coefficient of determination of 0.56.

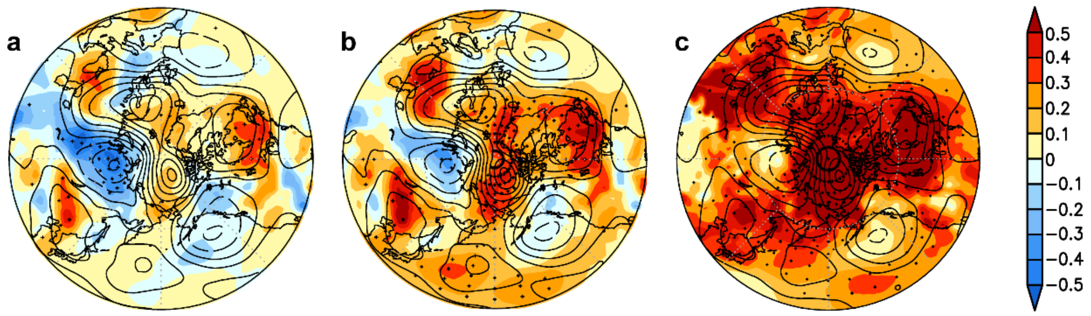

Supplementary Figure 15 | Observed and simulated changes in atmospheric circulation and lower-tropospheric temperature. a–c Linear trends of Z500 (contour, 25 m interval) and 1000–850 hPa temperature (shading, °C per decade) over the period 1979–2016 for forced-nudged simulation (**a**), slab-nudged simulation (**b**) and observations (**c**). Shading with stippling indicates statistical significance at the 5% level in all plots.

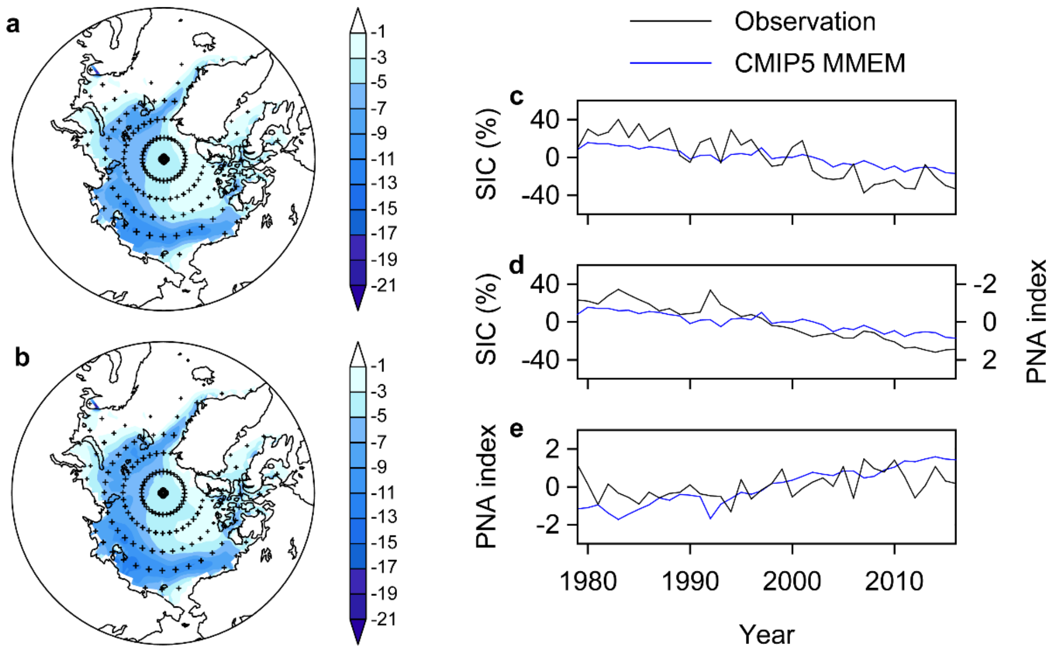

Supplementary Figure 16 | Arctic SIC responses to PNA changes in the CMIP5 multi-model ensemble mean (MMEM). a, Linear trend (% per decade) of SIC over the period 1979–2016. **b**, SIC regressed onto the PNA index. **c**, Time series of the western Arctic SIC anomaly. **d,** Time series of the western Arctic SIC anomaly along with the PNA index. **e,** Time series of the PNA index. Shading with stippling indicates statistical significance at the 5% level in **a** and **b**. The 12 CMIP5[2] models include ACCESS1-0, ACCESS1-3, CCSM4, CESM1-CAM5, CNRM-CM5, CSIRO-Mk3-6-0, FIO-ESM, GISS-E2-H, HadGEM2-AO, IPSL-CM5A-MR, MIROC5 and MPI-ESM-LR.

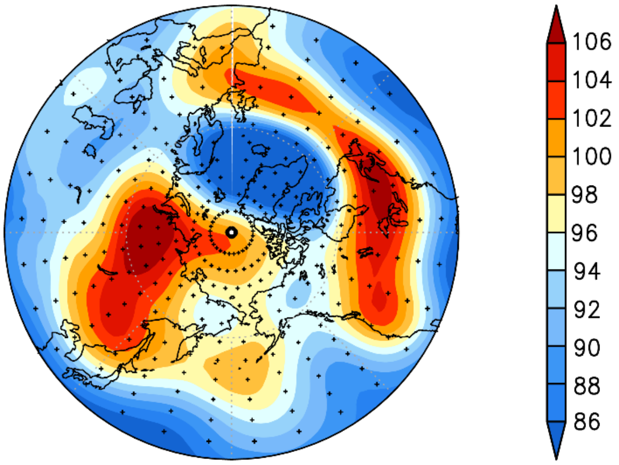

Supplementary Figure 17 | Projected changes in atmospheric circulation pattern. Difference in multi-model ensemble mean (MMEM) Z500 (m) between the RCP 8.5 simulation and the historical simulation from 12 Coupled Model Intercomparison Project Phase 5 (CMIP5)[2] models, calculated as 2070–2095 minus 1980–2005. Areas with significance at the $p < 0.05$ level are stippled. The MMEM projects an enhanced positive PNA-like pattern for the future period, relative to the historical period, with eastward and northward shifts in the centers of action[3].

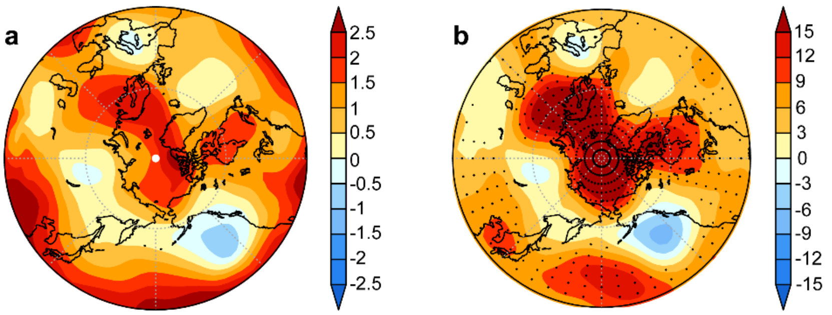

Supplementary Figure 18 | Simulated PNA pattern. a, Simulated pattern of the first Empirical Orthogonal Function (EOF1, 22.94 % of the total variance) of Z500. **b**, Z500 (m) regressed onto the standardized EOF1 time series shown in Fig. 5**c**. Shading with stippling indicates statistical significance at the 5% level in **b**.

References

1 Ding, Q. *et al.* Influence of high-latitude atmospheric circulation changes on summertime Arctic sea ice. *Nat. Clim. Change* **7**, 289 (2017).

2 Taylor, K. E., Stouffer, R. J. & Meehl, G. A. An overview of CMIP5 and the experiment design. *Bull. Am. Meteorol. Soc.* **93**, 485-498 (2012).

3 Liu, Z., He, X., Ma, W. & Wang, Y. Robust increases in extreme Pacific North American events under greenhouse warming. *Geophys. Res. Lett.* **47**, doi:https://doi.org/10.1029/2019GL086309 (2019).
